# Molecular Interaction and Solubilization Efficiency of Neohesperidin in Ternary Systems with Hydroxypropyl-β-cyclodextrin and Meglumine

**DOI:** 10.3390/foods13193143

**Published:** 2024-10-01

**Authors:** Na Xia, Yanquan Liu, Dan Gao, Siming Zhu

**Affiliations:** 1 College of Life and Geographic Sciences, Kashi University, Kashi 844000, China; conniexn@126.com (N.X.); liuyanquan1823@163.com (Y.L.); 2 Key Laboratory of Biological Resources and Ecology of Pamirs Plateau of Xinjiang, Kashi University, Kashi 844000, China; 3 School of Food Science and Engineering, South China University of Technology, Guangzhou 510641, China; 4 Institute of Chinese Materia Medica, China Academy of Chinese Medical Sciences, Beijing 100050, China; dgao@icmm.ac.cn

**Keywords:** Neohesperidin, HP-β-CD, meglumine, complex, solubility

## Abstract

The solubilization of poorly water-soluble natural bioactive compounds remains a significant challenge. This study aims to design a ternary inclusion system to enhance the solubility of the poorly water-soluble compound Neohesperidin (NH). Soluble ternary cyclodextrin complexations (t-CDs) containing NH, 2-hydroxypropyl-β-cyclodextrin (HP-β-CD), and meglumine (MEG) were prepared and optimized. The optimized t-CDs were further characterized using Scanning Electron Microscopy (SEM), Powder X-ray Diffraction (PXRD), Differential Scanning Calorimetry (DSC), Fourier Transform Infrared Spectroscopy (FTIR), Nuclear Magnetic Resonance (NMR), and molecular docking (MD) techniques. The results suggested that NH formed was associated with MEG through hydrogen bonds with MEG, and was subsequently incorporated into the hydrophobic cavity of HP-β-CD, which may be a key factor in improving its solubility. The solubility of NH in water at 37 °C increased significantly from 0.16 mg/mL to 5.81 mg/mL in the optimized t-CDs (NH/MEG/HP-β-CD).

## 1. Introduction

Neohesperidin is an important natural flavonoid glycoside with a range of beneficial biological and pharmacological activities. However, limited solubility, susceptibility to degradation under extreme conditions, and low bioavailability limit its use as a nutrient or drug [1]. In previous studies, our research group selected hydroxypropyl-β-cyclodextrin (HP-β-CD) due to its excellent aqueous solubility, enhanced thermal stability, high loading capacity, and low toxicity, as a drug carrier for neohesperidin [1]. Previous studies have identified the issue of requiring a high dosage of HP-β-CD during its use. For example, Li Xiao xi et al. artificially increased the solubility of the insoluble drug ST-246 to 20 mg/mL, increasing the concentration of HP-β-CD to 40% (*w*/*v*) [2].

To enhance the dissolution capacity of cyclodextrin, researchers have introduced additional reagents to develop ternary systems. Examples include polyethylene glycol 6000 (PEG6000), hydroxypropyl methylcellulose (HPMC) [3], Soluplus (polyvinyl alcohol-vinyl acetate-polyethylene glycol graft copolymer) [4,5], triethanolamine (TEA) [6], chitosan [7], and organic acids [8]. The addition of a third reagent reduces the required amount of cyclodextrin, as well as the toxicity, cost, and volume of the prepared solution [9]. Therefore, ternary cyclodextrin systems are a promising approach for enhancing the apparent solubility of insoluble drugs and have attracted significant interest in recent years [10]. However, drug/CD/organic base ternary complexes have been rarely reported. N-acetylglucosamine, also known as meglumine (MEG), is composed of polyhydroxylated organic amines and polyhydroxyl groups [11]. This unique structure endows meglumine with excellent water solubility and biocompatibility, making it a promising candidate for widespread use in pharmaceutical formulations. By forming drug-polymer complexes, MEG effectively increases the spatial separation between drug molecules, thereby lowering their lattice energy and improving their water solubility. Furthermore, MEG can also establish stable complexes with drug molecules through hydrogen bonding, which further facilitates drug dissolution [12].

In this study, we designed and prepared a ternary inclusion complex (NH-MEG-CD) consisting of NH, HP-β-CD, and MEG. Following freeze-drying, the samples were characterized by SEM, PXRD, DSC, FTIR, and NMR. To further elucidate the interactions within the ternary inclusion complex, molecular docking simulations were employed to study the binding modes and verify the interaction mechanism. The objective is to improve the solubility of NH, address the technical challenge of NH’s insolubility, and provide a theoretical foundation for its large-scale production in food and pharmaceutical preparations.

## 2. Materials and Methods

### 2.1. Materials

NH (purity ≥ 98%) was obtained from Shandong Benyue Biological Technology Co., Ltd. (Dongying, China). HP-β-CD (purity ≥ 99.5%), MEG, polyvinylpyrrolidone (PVP-12), polyethylene glycol 400 (PEG400), sodium carboxymethyl cellulose (CMC), tartaric acid, citric acid, and sodium salicylate were obtained from Macklin Biochemical Co. Ltd. (Shanghai, China). Chromatographic methanol was supplied by Sigma-Aldrich Co., Ltd. (St. Louis, MS, USA). All reagents used were of Analytical Reagent (A. R.) grade.

### 2.2. Co-Solvent Screening

Various organic acids, amines, and polymers were screened to enhance the solubility of neohesperidin without the use of HP-β-CD, and an appropriate co-solute was identified. Tartaric acid, citric acid, sodium salicylate, MEG, PVP-12, PEG400, CMC, and Poloxamer-188 were selected as co-solutes to evaluate their solubilizing effects on neohesperidin [13].

### 2.3. Determination of Co-Solvent Concentration

Distilled water was used to prepare co-solvent solutions with concentrations ranging from 0.2 to 1.6%. Excess NH was added to small conical flasks containing various concentrations of the co-solvent solutions. The solutions were placed on a constant temperature shaker at 25 °C and agitated for 24 h at a speed of 120 rpm. The dissolution of NH in different co-solvent concentrations was measured using high-performance liquid chromatography (HPLC) with a Shimadzu LC-10A liquid chromatograph (Shimadzu, Kyoto, Japan).

We used Wondasil C18 analytical columns (250 mm × 4.6 mm, 5 µm particle size) with a mobile phase consisting of methanol and formic acid. Gradient elution was performed with a UV detector set at a wavelength of 283 nm. The operating temperature of the reverse-phase column was maintained at 35 °C, with an injection volume of 20 µL. Each gradient program lasted 30 min. The detailed gradient elution program is presented in Table 1.

### 2.4. Phase Solubility Study

The phase solubility was determined using the method described by Higuchi [12] and Connors [14]. Samples were analyzed at 284 nm using HPLC. The inclusion rate (CE) was defined as the dissolution efficiency of CD for guest molecules, referring to the ability of CD to incorporate drugs into its hydrophobic cavity and form a complex with hydrophobic molecules. The effect of meglumine on solubility was determined from the phase solubility diagram. The stability constants (*Kc*) of the binary and ternary systems were calculated using the slope of the phase solubility diagram in water. In general, comparing CE values is more convenient than *Kc* values, as CE is less sensitive to errors in the intrinsic solubility of the drug and aids in selecting optimal conditions for drug complexation with CD [14,15].

The intrinsic solubility (*S_O_*) of a drug is calculated using the following formula:

(1) $$Kc=\frac{Slope}{S_{O}(1-Slope)}$$

where *Kc* is the stability constant, and *So* represents the solubility of NH without HP-β-CD and MEG. The complexation efficiency (CE) value was calculated using the following formula:

(2) $$CE=\frac{Slope}{\left(1-Slope\right)}$$

### 2.5. Preparation of NH Inclusion Complex

Inclusion compounds were prepared using the solvent co-evaporation method (CE). It has been reported that inclusion compounds derived from this technique exhibit the highest levels of solubility [16].

Solvent Co-Evaporation Method: During the preparation of the inclusion complexes, the molar ratio of NH to HP-β-CD was maintained at 1:1. Aqueous solutions of HP-β-CD and ethanol solutions of NH were prepared separately. The aqueous phase was stirred using a magnetic agitator and gradually added to the organic phase. After thoroughly mixing, the mixture was heated at a high temperature to evaporate the solvent. Using the same method, the same molar amount of MEG was added to the HP-β-CD solution to prepare the ternary inclusion complex, similar to the binary inclusion complex. The sample was then freeze-dried and stored in a desiccator.

### 2.6. Saturation Solubility Study

The excess inclusion compound was placed in a conical flask containing 10 mL of distilled water and shaken in a constant temperature water bath shaker at 120 rpm for 24 h at ambient temperature. After equilibrium was reached, the samples were filtered using a 0.45 μm microporous membrane. The NH content of the inclusion compounds was then determined by HPLC [17].

### 2.7. Characterization of Ternary Inclusion Complex

#### 2.7.1. Fourier Transform Infrared Spectrometer (FTIR)

To thoroughly investigate the changes in functional groups of NH and the NH clathrate following the addition of a co-solvent, a series of samples were prepared. This process involved carefully blending NH, HP-β-CD, meglumine, binary inclusion complexes, and ternary inclusion complexes with 100 mg of KBr powder. The samples were then characterized by FTIR (Vector 33, Bruker, Ettlingen, Germany) with a resolution of 4 cm^−1^ and a scanning range of 4000–500 cm^−1^. The method followed was the same as described by Wang et al. [17,18].

#### 2.7.2. Powder X-ray Diffraction (PXRD)

The PXRD patterns of NH, MEG, and inclusion complex samples were analyzed using a multi-position automatic X-ray diffractometer (D8 ADVANCE, Bruker, Germany) to examine changes in characteristic peaks before and after the formation of the NH inclusion complex. The method followed was the same as described by Wang et al. [17]. Major peak and intensity data were collected and analyzed using Origin 9.1 software. The crystallinity was determined by comparing the height of representative peaks in the diffraction pattern of the ternary inclusion complex with that of NH.

#### 2.7.3. Thermal Analyses

Approximately 3.0 mg of each sample, including neohesperidin, HP-β-CD, meglumine, their binary inclusion complex, and ternary inclusion complex, were accurately weighed into a crucible. Differential thermogravimetric analysis was then conducted using a differential scanning calorimeter (STA449C, NETZSCH, Waldkraiburg, Germany). Nitrogen was used as a protective gas, with the heating rate set at 10 °C/min and a temperature range of 50 °C to 500 °C. Each sample was analyzed three times to ensure reproducibility and accuracy.

#### 2.7.4. SEM

An appropriate amount of NH, HP-β-CD, MEG, NH-HPβCD, and NH-MEG-HPβCD inclusion compound were taken, and the samples were treated with gold sputter coating at an accelerated voltage of 2.0 kV (EVO18 ZEISS, Oberkochen, Germany). The appearance of the sample was scanned to observe the morphology of the sample before and after inclusion.

#### 2.7.5. ^1^H NMR

NH, HP-β-CD, MEG, binary inclusion complex, and ternary inclusion complex samples were evaluated with ^1^H NMR (Bruker II 400 NMR Spectrometer) to determine complex formation. DMSO-d6 was used as the solvent for NH, and other samples were dissolved in D2O.

#### 2.7.6. In Vitro Dissolution Test

The dialysis method was used to study the dissolution of NH and its binary and ternary inclusion complexes [19]. The binary inclusion compound containing cyclodextrin and the ternary inclusion compound containing MEG and cyclodextrin were placed in 50 mL of phosphate buffer (pH 6.8) and gently shaken at 37 ± 0.5 °C (100 rpm). At predetermined intervals of 5, 10, 15, 30, 45, 60, 75, and 90 min, 1 mL of the leaching solution was collected, and the same volume of fresh phosphate buffer was added to maintain a constant volume. The samples were analyzed by HPLC. The experiment was repeated three times, and the release rate was calculated.

#### 2.7.7. Molecular Model Construction

Autodock 4.2 was used for molecular docking, with the HP-β-CD and NH docking complex serving as the receptor and MEG as the ligand. The AutoDockTools 1.5.6 program was employed to preprocess the cyclodextrins and small molecules, saving them in pdbqt format. During the docking procedure, the cyclodextrin complex was treated as rigid, while the meglumine molecule was designated as flexible. Molecular docking was performed using the Autodock 4.2 software, with a total of 100 independent docking runs conducted. Cluster analysis was then performed with a truncation value of 2 Å. The number of clusters and the minimum binding free energy for each cluster were recorded. The interaction between NH and HP-β-CD in the inclusion complexes was assessed by calculating the interaction energy or binding affinity. The Prime MM-GBSA module (version 4.5) was used to compute the energy based on a comparison between the solvent-accessible area and the energy of an isolated molecule [20]. Two potential energy calculation methods were applied: the first, known as the “energy difference mode”, involved calculating the energy for the receptor, then the ligand, and finally the complex, with a focus on the relaxation of ligand and receptor binding. The energy difference was denoted as:

DelE = Ecomplex – Eligand − Ecyclodextrin(3)

The second model referred to as “interaction energy model”, which calculates the interaction energy between the receptor and the ligand. This energy was determined based on the interactions between the ligand atoms and receptor atoms, excluding any contributions from the ligand or receptor atoms themselves.

### 2.8. Statistical Analysis

The resulting data were graphed using Origin 9.0 and analyzed using IBM SPSS Statistics 26. A *t*-test or one-way ANOVA was performed to determine any significant differences, with a *p*-value of <0.05 considered statistically significant. Data were reported as the mean ± standard deviation (SD).

## 3. Results

### 3.1. Selection of Co-Solvent

**Figure 1:**
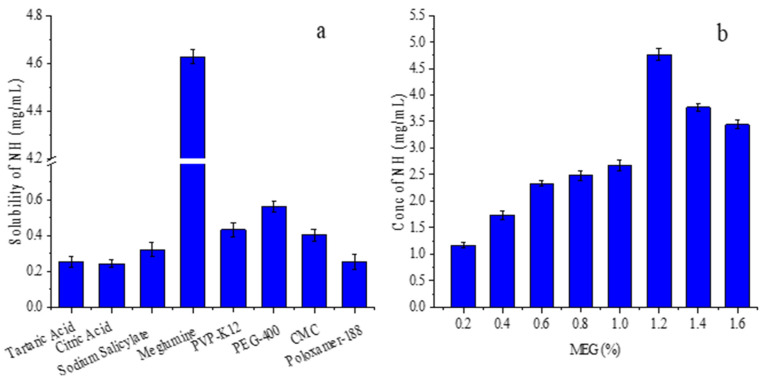
Solubility of NH in (**a**) series of co-solvents; (**b**) various concentrations of meglumine solutions.

The screening results of NH co-solvents are shown in Figure 1a. Among the co-solvents tested, including organic acids, amines, and polymers, meglumine demonstrated the most significant solubilizing effect, increasing the maximum solubility of NH to 4.76 ± 0.11 mg/mL. The solubility of NH increased with rising MEG concentration, up to 1.2%, after which no significant further increase in solubility was observed, as shown in Figure 1b. Therefore, 1.2% MEG was selected as the third component for the phase solubility study and the preparation of the ternary complex.

### 3.2. Study on Phase Solubility

**Figure 2:**
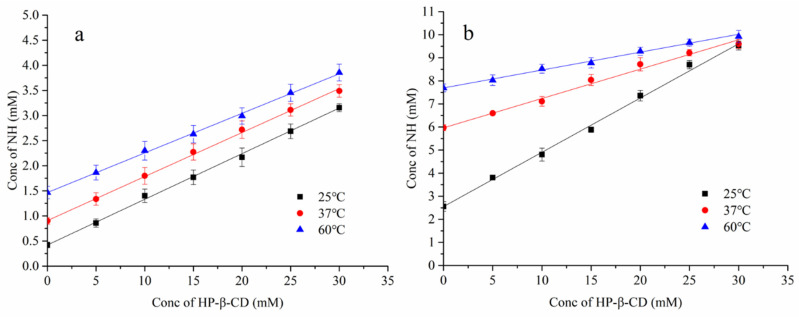
Phase-solubility diagrams of inclusion complexes formed by NH-CD (**a**) and NH-MEG-CD (**b**) at different temperatures.

Figure 2a,b present the phase solubility of binary inclusion complexes (NH-CD) and ternary inclusion complexes (NH-MEG-CD) in the presence of MEG, respectively. The results indicate that the solubility of the drug increases with the concentration of HP-β-CD. When the concentration of HP-β-CD ranged from 0 to 30 mmol/L, there was a linear relationship between the absorbance of NH and the concentration of HP-β-CD. The linear equation, correlation coefficient (R^2^), and stability constant (*Kc*) are presented in Table 2. Figure 2 shows that ternary inclusion complexes (NH-MEG-CD), with MEG as a co-solvent, have greater solubility for NH than binary inclusion complexes (NH-CD).

The *Kc* value is often used to reflect the inclusion ability and stability between cyclodextrin and guest molecules, and temperature typically has a significant impact on the *Kc* value [12,21]. Table 1 shows that the stability constant (*Kc)* of the ternary inclusion complex (NH-MEG-CD) is higher than that of the binary inclusion complex (NH-CD) at 25 °C and 37 °C. This indicates that the presence of MEG as a third component enhances the interaction between NH and HP-β-CD, thereby improving the solubility of NH-MEG-CD complex. The increase in the stability constant can be attributed to the cyclodextrin (CD) cavity promoting the release of high-energy water molecules by establishing interactions with the drug, such as hydrogen bonds, hydrophobic interactions, and van der Waals forces. This enhances the inclusion ability of the CD cavity for the drug [12]. Therefore, compared to binary inclusion complexes, the use of a co-solvent in the preparation of ternary inclusion complexes can further improve the solubility of drugs.

In addition, as shown in the phase solubility graphs in Figure 2a,b at different temperatures, it was observed that the solubility of both binary and ternary inclusion complexes increased with rising temperature. This may be attributed to the inherent property of NH to become more soluble under heating conditions and the release of high-energy water molecules bound to the cyclodextrin cavity at elevated temperature [22,23]. The *Kc* value of both complexes decreased with increasing temperature, which differs from some previous studies [24]. At 25 °C and 60 °C, the inclusion constants of NH-CD and NH-MEG-CD decreased from 826.75 to 331.17 L·moL^−1^ and from 2558.28 to 325.36 L·moL^−1^, respectively, indicating that the number of inclusion complexes decreased with rising temperature. This may be due to the disruption of hydrogen bonds between the inclusion complexes during heating, resulting in a reduction in hydrogen bond strength [21,22].

### 3.3. Saturation Solubility

**Figure 3:**
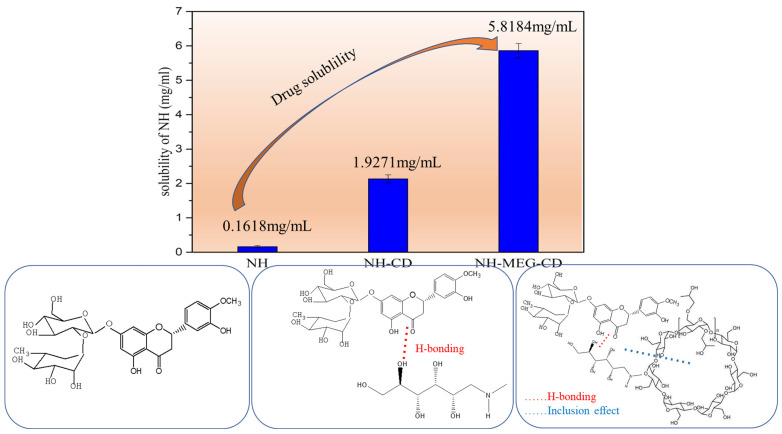
Saturated solubility of NH and its binary and ternary complexes in water at 37 ± 2 °C.

The saturation solubility results of NH and its binary and ternary inclusion complexes in water are shown in Figure 3. NH forms binary and ternary inclusion complexes with HP-β-CD, which increases its water solubility. The solubility of NH in the NH-MEG-CD ternary inclusion complexes increased more than that in NH-CD binary inclusion complexes. The solubility of pure NH in water was 0.16 ± 0.03 mg/mL, and the solubility of NH in NH-CD was 1.92 ± 0.12 mg/mL, an increase of 12 times. The solubility of NH-MEG-CD was 5.81 ± 0.21 mg/mL, an enhancement of 36 times.

It has been previously reported that co-solvents, such as weak acids, bases, and hydrophilic polymers, increase the water solubility of drugs through surface interactions with drug inclusion complexes [25,26,27]. In addition, hydrogen bonding, electrostatic forces, hydrophobic interactions, and van der Waals forces between the drug and the co-solvent play a key role in enhancing solubility. It has been reported that co-solvents, such as weak acids or bases like citric acid and meglumine, can form ternary complexes with cyclodextrin through hydrogen bonding, salting, and electrostatic interaction with drugs [28]. Polymer co-solvents interact with the hydrophobic part of HP-β-CD their hydrophobic regions to form supramolecular ternary complexes. The hydrophilic portion is oriented toward the surface of the cyclodextrin complex, reducing surface tension and thereby increasing water solubility.

### 3.4. Physicochemical Characterization of Inclusion Complex

#### 3.4.1. FTIR Analysis

**Figure 4:**
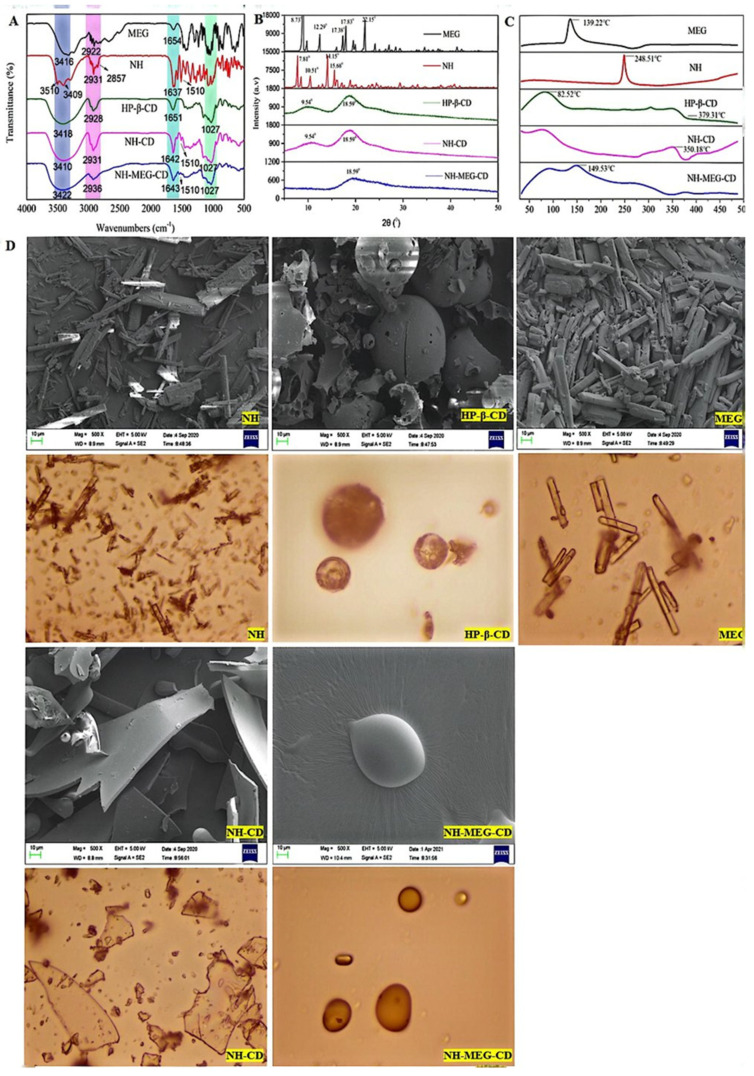
(**A**) FTIR spectra: MEG, NH, HP-β-CD, NH-CD and NH-MEG-CD; (**B**) XRD spectra of MEG, NH, HP-β-CD, NH-CD and NH-MEG-CD; (**C**) DSC thermograms of MEG, NH, HP-β-CD, NH-CD and NH-MEG-CD; (**D**) SEM photographs and Photomicrographs of NH, HP-β-CD, MEG, NH-CD and NH-MEG-CD.

As shown in Figure 4A, NH, MEG, and HP-β-CD each exhibit specific infrared absorption peaks. The FT-IR spectra of NH show two characteristic -OH peaks at 3510 and 3409 cm^−1^. The peak at 2931 cm^−1^ corresponds to the stretching vibration of the C-H bond on the B-cyclomethoxy group of NH. The peak at 1637 cm^−1^, corresponds to the stretching vibration of the cyclocarbonyl C=O. The peak at 1510 cm^−1^ is assigned to the stretching vibration of the C=C bond in the benzene ring skeleton. The FT-IR spectra of MEG showed a characteristic -OH peak at 3416 cm^−1^. The stretching vibration peak of alkane C-H appeared at 2922 cm^−1^, and a variable-angle vibration peak of -NH was observed at 1654 cm^−1^. The FT-IR spectra of HP-β-CD showed a broad -OH band at approximately 3418 cm^−1^. A characteristic peak at 1650 cm^−1^ corresponded to the H-O-H deformation band of water molecules attached to HP-β-CD. The stretching vibration peak of C-O-C appeared at 1027 cm^−1^.

Compared with HP-β-CD, the FT-IR spectra of NH-CD and NH-MEG-CD showed shifts from 3418 cm^−1^ to 3410 cm^−1^ and 3422 cm^−1^, respectively, indicating changes in peak position and the width of the -OH characteristic peak. The peak at 2928 cm^−1^ for HP-β-CD shifted to 2931 cm^−1^ for NH-CD and 2936 cm^−1^ for NH-MEG-CD, suggesting that both -OH and alkyl C-H groups were involved in the bonding. In addition, the carbonyl peak of NH shifted from 1637 cm^−1^ to 1642 cm^−1^ and 1643 cm^−1^ in NH-CD and NH-MEG-CD, respectively, indicating its participation in the interaction. The -OH characteristic bimodal peaks of NH at 3510 cm^−1^ and 3409 cm^−1^ disappeared in both NH-CD and NH-MEG-CD, and the stretching vibration peaks of C=C at 1510 cm^−1^ weakened, suggesting that the aromatic ring was also involved in π-π interactions. This may be due to NH being incorporated into the HP-β-CD cavity, resulting in the reduction or disappearance of absorption intensity.

#### 3.4.2. Powder XRD

The X-ray diffraction patterns of NH, MEG, HP-β-CD, NH-CD, and NH-MEG-CD powders are shown in Figure 4B. NH and MEG exhibited prominent crystal diffraction peaks, indicating high crystallinity. The diffraction peaks of NH at 2*θ*° were 7.81°, 8.51°, 10.51°, 14.15°, and 15.68°, respectively. The diffraction peaks of MEG at 2θ°were 8.73°, 9.71°, 12.29°, 17.38°, 17.83°, and 22.15°, respectively. HP-β-CD exhibited two broad, weak diffraction peaks at 2*θ*° values of 9.54° and 18.59°, indicating its amorphous structure. NH-CD also showed an amorphous structure, with only two weak diffraction peaks corresponding to HP-β-CD at 2θ° values of 9.54° and 18.59°. NH-MEG-CD displayed no significant crystal diffraction peaks, suggesting that the drug forms an amorphous structure [29]. The transition of NH from a crystalline to an amorphous state in the inclusion complex contributes to improved drug solubility.

#### 3.4.3. DSC Analysis

When drug molecules are embedded in the intramolecular cavity of cyclodextrin, their melting, boiling, and sublimation points typically shift to another temperature, decrease to some extent, or completely disappear [30]. As shown in Figure 4C, the DSC curve indicates that MEG has a distinct endothermic peak at its melting point of 139.2 °C. NH also exhibited a sharp endothermic peak at 248.5 °C, reflecting its crystalline structure. HP-β-CD displayed a broad endothermic peak at 82.52 °C, which was attributed to the loss of water from the lumen of HP-β-CD.

Comparing the peaks of various complexes with those of NH reveals significant differences in both the melting points and the intensity of the peaks, confirming the hypothesis of complex formation. The changes in the endothermic peaks were attributed to the loss of water molecules, the formation of the complex, the decrease in crystallinity, and the increase in the amorphous properties of the sample. Compared to NH, the characteristic peak of the binary complex (NH-CD) changed significantly, with only a small endothermic peak observed at 350.18 °C, indicating that the crystallinity of NH in HP-β-CD decreased significantly, and NH transformed into an amorphous state. In the ternary inclusion complex NH-MEG-CD, the DSC absorption peak of NH completely disappeared, and only the absorption peak of MEG appeared at 149.53 °C. The absence of a melting peak indicated that the drug existed in an amorphous form [31]. This suggests that the interaction between NH and HP-β-CD was enhanced in the presence of MEG. Therefore, based on the shifts in the endothermic peaks, it can be concluded that a strong interaction occurred between NH and HP-β-CD during the formation of the inclusion complex. As a result, NH may transform from a crystalline structure to an amorphous form due to the formation of the inclusion complex.

#### 3.4.4. SEM Analysis

Figure 4D shows the surface morphology of MEG, NH, HP-β-CD, NH-CD, and NH-MEG-CD. MEG exhibited as columnar acicular crystals, while NH exhibited irregular acicular crystals. HP-β-CD observed as amorphous spherical particles, hollow inside, with numerous round holes on the surface. During the inclusion process with NH, the porous spherical morphology of HP-β-CD in NH-CD disappeared, forming a smooth, regular tile-like structure. The powder of NH-MEG-CD exhibited irregular particles, and the crystal structures of both NH and MEG disappeared. These observations are consistent with the PXRD results, indicating that NH and MEG may be non-uniformly distributed within the cavity of HP-β-CD.

#### 3.4.5. ^1^H NMR Measurements

**Figure 5:**
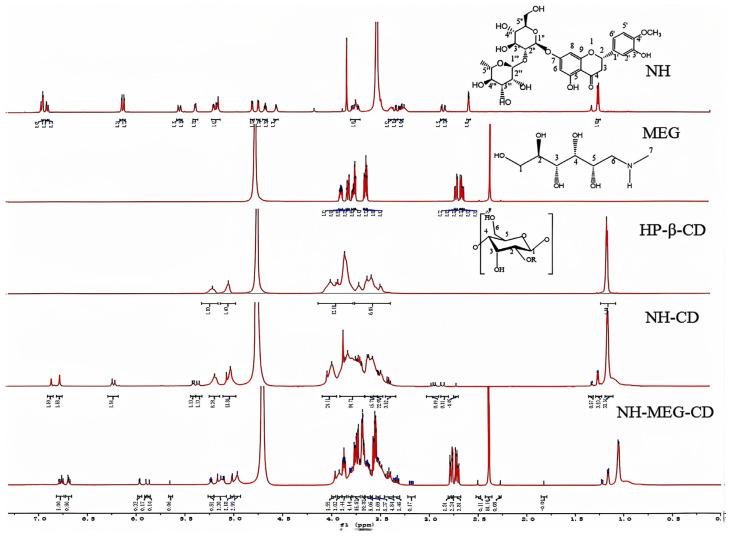
Expanded NMR spectra of NH, MEG, HP-β-CD, NH-CD and NH-MEG-CD.

The ^1^H NMR signal spectra of NH, HP-β-CD, MEG, and the binary and ternary complexes in deuterated solvents were compared. Figure 5 confirms the formation of NH-MEG-CD ternary inclusion complexes. The structure of the inclusion complexes can be determined based on the chemical shifts observed in the binary and ternary inclusion complexes [32].

The ^1^H NMR spectra of the complex can be attributed to changes in the HP-β-CD cavity to accommodate the guest drug molecule. The ^1^H NMR spectra of the binary and ternary supramolecular complexes showed multiple peaks in the δ1.0–7.0 ppm range, with deviations observed in HP-β-CD and NH, indicating interaction with the drug NH. As shown in Table 3, each peak of NH exhibited a chemical shift induced by HP-β-CD. Compared to the binary inclusion complex, the proton chemical shifts of NH in the ternary inclusion complex at H-8, H-6, H-6′, H-2′, and H-5′ were smaller, with Δδ values of 0.259, 0.243, −0.007, −0.256, −0.177 ppm, respectively. The results indicated that the aromatic ring group of NH was largely encapsulated by the hydrophobic cavity of HP-β-CD [33], leading to an increase in NH’s solubility. Additionally, the small chemical shift changes in the corresponding peaks of the cyclopropyl ring, along with the presence of multiple peaks in the δ1.0–1.3 ppm range, suggest significant hydrophobic interactions between NH and the cyclopropyl ring. These interactions may have contributed to the change in solubility. The NMR spectra of the ternary complexes revealed significant changes in the chemical shift of MEG, with distinct material peaks observed in the range of 2.0 to 4.0 ppm. The decrease electron-absorbing capacity of the carbonyl group in NH led to the displacement of H-6a and H-6b, indicating the presence of intermolecular hydrogen bonding between the carbonyl group in NH and MEG [2]. The proton displacement in the ternary inclusion complex of HP-β-CD may also be attributed to the formation of multiple hydrogen bonds between the amino group on the main chain of MEG and the hydroxyl group of glucopyranose. It can be concluded that the addition of MEG acts as a bridge between NH and HP-β-CD, playing a key role in enhancing the solubility of NH [34]. By comparing the ^1^H NMR spectra of the binary and ternary complexes, the presence of multiple peaks in the δ2.5–4.0 ppm and 1.0–1.3 ppm range in the supramolecular ternary complexes may explain the enhanced solubility of the ternary complexes compared to the binary complexes.

#### 3.4.6. In Vitro Dissolution Analysis

**Figure 6:**
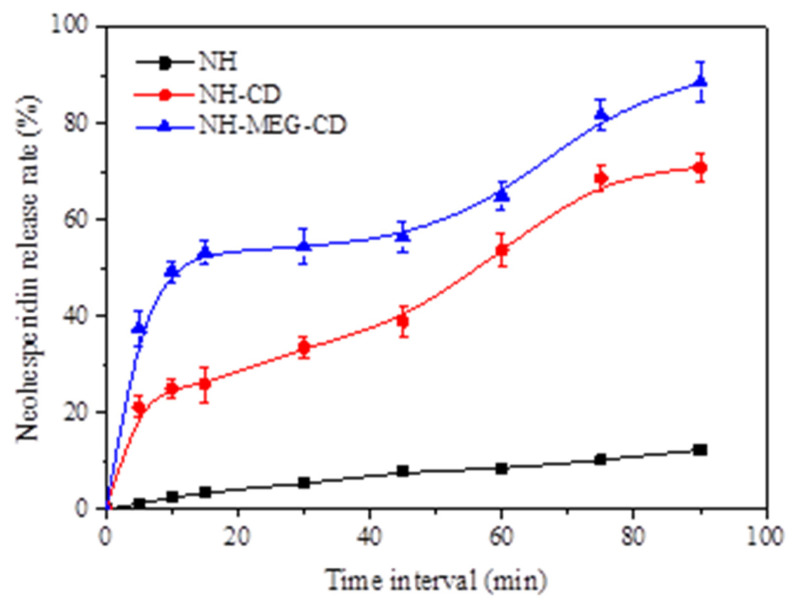
In vitro dissolution profile of pure NH and other complexes in pH 6.8 phosphate buffer.

The in vitro release behavior of NH and its inclusion complexes was studied using an in vitro dissolution experiment, as shown in Figure 6. The dissolution study was conducted in phosphate buffer at pH 6.8. NH release was 2.45 ± 0.36% in the first 10 min, and the maximum drug release reached 12.33 ± 0.49% at 90 min. The low solubility of NH in phosphate buffer at pH 6.8 results in poor release of pure NH. The dissolution rate of the binary and ternary inclusion complexes of HP-β-CD increased significantly. The NH release for NH-CD and NH-MEG-CD was 25.11 ± 1.86% and 49.32 ± 2.15% at 10 min, and 70.84 ± 2.68% and 88.61 ± 3.99% at 90 min, respectively. The order of NH release is NH < NH-CD < NH-MEG-CD. This was consistent with the saturation solubility results.

The release rate of NH-CD was higher than that of pure NH, primarily due to the formation of a binary inclusion complex between NH and HP-β-CD in the dissolution medium [35]. Compared to pure NH, the drug dissolution of the binary inclusion complex and the ternary inclusion complex increased by 5.74 and 7.18 times, respectively. The dissolution rate of ternary complex was significantly higher than that of the binary complex, likely due to the addition of the co-solvent MEG. The addition of MEG increased the hydrophilicity of the NH-CD complex and enhanced its hydrogen bonding capacity [30]. PXRD analysis showed that the drug release properties of the complex prepared with HP-β-CD were improved, as the formation of the inclusion complex reduced the crystallinity of NH.

#### 3.4.7. Computational Simulation of NH Inclusion Complex

The supramolecular ternary complexes of NH, HP-β-CD, and MEG were formed by attaching MEG to the binary inclusion complexes of NH and HP-β-CD. Figure S1 shows the docking result with the lowest energy. By analyzing all the docking conformations, no separation of NH and MEG from the cyclodextrin cavity was observed, indicating that NH and MEG molecules can form stable ternary inclusion complexes with cyclodextrin. The docking results showed that MEG was located in the hydroxypropyl-substituted region of HP-β-CD. The hydroxyl group of MEG forms a hydrogen bond with the carbonyl group of NH, with a bond length of 3.2 Å. The side chain carbon atoms of MEG stabilize the supramolecular ternary complex by forming van der Waals interactions with the benzene ring of NH. Thus, MEG stabilizes the supramolecular ternary inclusion complex through non-bonding interactions with both NH and HP-β-CD. The correlation between the binding energy and the stability constant of the ternary supramolecular and binary inclusion complexes was calculated.

As shown in Table 4, the energy calculation results for the binary and ternary inclusion complexes of NH were accepted. Using the energy difference model, it was calculated that both the binary inclusion complexes and the ternary supramolecular inclusion complexes exhibit high binding affinity. Compared to the binary inclusion complex, the ternary supramolecular inclusion complex showed the highest binding affinity between NH, HP-β-CD, and MEG. Total energy was −29.18 kcal/moL, the van der Waals energy was −55.47 kcal/moL, and the electrostatic energy was −19.24 kcal/moL. In addition, the interaction energy of NH with the host in binary and ternary inclusion complexes was calculated using the interaction energy model. As shown in Table 3, both the binary inclusion complex and the ternary supramolecular inclusion complex exhibited higher total binding energy, consistent with the results from the energy difference model. The contribution of van der Waals interactions to the stability of binary and ternary supramolecular inclusion complexes was greater than that of electrostatic interactions.

## 4. Conclusions

In this study, we prepared the NH, HP-β-CD, and MEG ternary complex and demonstrated that meglumine is an effective auxiliary substance that improves the solubility of NH through the inclusion phenomenon. First, the apparent stability constants *Kc* and CE of HP-β-CD were increased by addition of MEG, indicating the formation of a stable inclusion complex with HP-β-CD. The molecular docking model showed that MEG forms a bond bridge between NH and HP-β-CD through hydrogen bonding, van der Waals forces, and electrostatic interactions, thereby improving the stability of the inclusion complex. The inclusion complexes of NH-CD and NH-MEG-CD significantly enhance the solubility of NH. Compared to the relatively low solubility of NH at 0.16 mg/mL (at 37 °C), the solubility of the NH-CD and NH-MEG-CD complexes is 1.92 mg/mL and 5.81 mg/mL, respectively, representing 5.74-fold and 7.18-fold increases over the solubility of free NH.

Finally, through SEM, PXRD, DSC, FTIR, and NMR analysis, it was confirmed that hydrogen bonding and the inclusion effect were the primary factors promoting the solubility of the inclusion complex. Additionally, this system can be applied to other poorly water-soluble drugs with similar physical properties, providing a theoretical basis for the large-scale production and application of NH and related derivatives.

## Figures and Tables

**Table 1:** Gradient elution program for neohesperidin content measurement.

Time (min)	Flow Rate (mL/min)	Percent of Mobile Phase Substances (%)	
		A	B
0	1.000	80	20
20	1.000	0	100
25	1.000	0	100
26	1.000	80	20
30	1.000	80	20

A: 0.1% formic acid solution (in water); B: methanol.

**Table 2:** Phase solubility studies of binary and ternary systems.

	Tem (°C)	Linear Regression Function	R^2^	K/(L·moL^−1^)
NH-CD	25	A = 0.0904c + 0.4231	0.9973	826.75
	37	A = 0.0886c + 0.9003	0.9983	600.82
	60	A = 0.0788c + 1.4654	0.9981	331.17
NH-MEG-CD	25	A = 0.2352c + 2.5518	0.9926	2558.28
	37	A = 0.1272c + 5.9644	0.9926	900.07
	60	A = 0.07752c + 7.6943	0.9897	325.36

**Table 3:** ^1^H-NMR chemical shift values of NH and inclusion complexes.

	Assignment	δ_free_ (ppm)	Δδ (δComplex − ΔFree Drug) (ppm)	
			NH-CD	NH-MEG-CD
NH	Rha-CH_3_	1.154	0.003	−0.008
	H-3	2.793	−0.008	−0.002
	OCH_3_	3.772	−0.011	−0.051
	Rha-H-1	5.011	−0.029	−0.049
	Glu-H-1	5.125	−0.012	−0.018
	OH	5.181	−0.038	−0.019
	H-2	5.392	−0.018	-
	H-8	6.122	0.064	0.259
	H-6	6.141	0.072	0.243
	H-6′	6.901	−0.087	−0.007
	H-2′	6.933	−0.012	−0.256
	H-5′	6.941	−0.009	−0.177
	OH-3′	9.092	−	−
	OH-5	12.025	−	−
MEG	H-1	3.8137	−	−0.003
	H-2	3.6981	−	−0.015
	H-3	3.6620	−	0.004
	H-4	3.6584	−	0.003
	H-5	3.5532	−	0.009
	H-6a	2.6108	−	0.156
	H-6b	2.5601	−	0.157
	H-7	2.2677	−	0.121
HP-β-CD	H-1	5.004	−0.043	−0.023
	H-2	3.644	0.033	−0.025
	H-3	3.938	−0.019	−0.066
	H-4	3.422	−0.012	−0.005
	H-5	3.793	−0.089	−0.074
	H-6	3.862	−0.051	−0.054
	CH_3_	1.063	−0.004	−0.006

**Table 4:** Embrace Energy Calculations.

Complex	Energy Difference Mode kcal/moL			Interaction Energy Mode kcal/moL		
	Del Total Energy	Del vdW	Del Electro	Total Energy	Vdw	Electrostat
binary	−23.6324	−48.8362	−16.5153	−149.2773	−112.369	−36.9083
ternary	−29.1803	−55.4772	−19.2427	−165.3823	−126.669	−38.7133

## Data Availability

The data presented in this study are included in the article and Supplementary Materials. Further inquiries can be directed to the corresponding authors.

## Supplementary Materials

The following supporting information can be downloaded at: https://www.mdpi.com/article/10.3390/foods13193143/s1, Figure S1. Docking calculation results and proposed favorable mode of NH-MEG-CD inclusion complex. (A) stick molecular representation; (B) top view from the wide rim; (C) side view. In yellow, hydrogen bonds.
