# Supplementary material for: Molecular Interaction and Solubilization Efficiency of Neohesperidin in Ternary Systems with Hydroxypropyl-β-cyclodextrin and Meglumine
Source: Foods. 2024 Oct 1;13(19):3143. doi: 10.3390/foods13193143 (PMC11475308; doi:10.3390/foods13193143)
Supplement: Supplementary file 1 [file foods-13-03143-s001.zip › foods-3134142-supplementary.pdf]

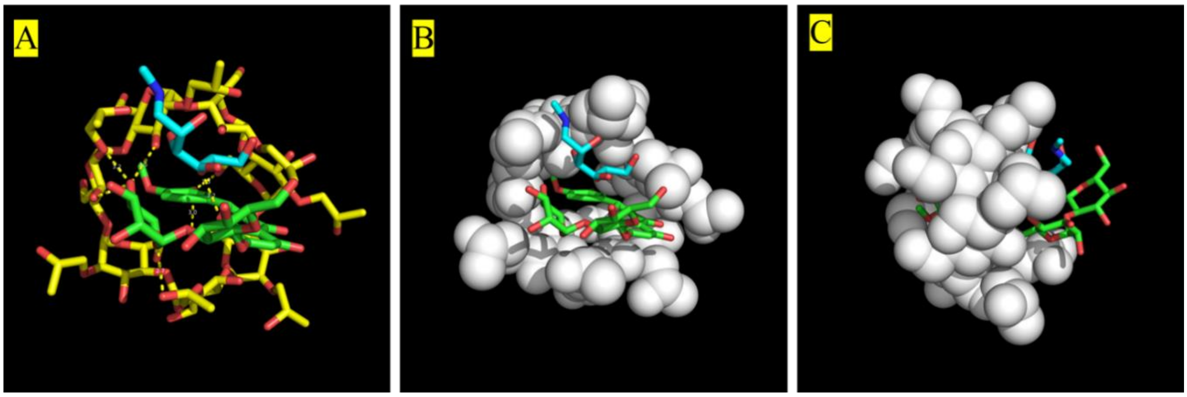

Figure S1. Docking calculation results and proposed favorable mode of NH-MEG-CD inclusion complex. (**A**) stick molecular representation; (**B**) top view from the wide rim; (**C**) side view. In yellow, hydrogen bonds.
